# Ex Vivo Evaluation of Insulin Nanoparticles Using Chitosan and Arabic Gum

**DOI:** 10.5402/2011/860109

**Published:** 2011-07-10

**Authors:** M. R. Avadi, A. M. M. Sadeghi, Naser Mohamadpour Dounighi, R. Dinarvand, F. Atyabi, M. Rafiee-Tehrani

**Affiliations:** ^1^Faculty of Pharmacy, Azad University of Medical Sciences, Tehran, Iran; ^2^Hakim Pharmaceutical Company, P.O. Box 11365-5465, Tehran, Iran; ^3^Razi Vaccine and Serum Research Institute, P.O. Box 3197619751 Karaj, Iran; ^4^Faculty of Pharmacy, Tehran University of Medical Sciences, Tehran, Iran

## Abstract

Polymeric delivery systems based on nanoparticles have emerged as a promising approach for peroral insulin delivery. The aim of the present study was to investigate the release of insulin nanoparticulate systems and ex vivo studies. The nanoparticles were prepared by the ion gelation method. Particle size distribution, zeta potential, and polydispersity index of the nanoparticles were determined. It was found that the nanoparticles carried positive charges and showed a size distribution in the range of 170–200 nm. The electrostatic interactions between the positively charged group of chitosan and negatively charged groups of Arabic gum play an important role in the association efficiency of insulin in nanoparticles. In vitro insulin release studies showed an initial burst followed by a slow release of insulin. The mucoadhesion of the nanosystem was evaluated using excised rat jejunum. Ex vivo studies have shown a significant increase in absorption of insulin in the presence of chitosan nanoparticles in comparison with free insulin.

## 1. Introduction

Since the parenteral administration is the only route of insulin delivery, alternative routes of administration (oral, nasal, rectal, pulmonary, and ocular) have been extensively investigated [1]. Insulin is a protein composed of two polypeptide chains which are covalently bound by disulfide bonds between cysteine residues. Although peroral application is considered as the most convenient route of drug administration especially in long-term treatment, it is well known that the bioavailability of insulin after oral application is very low due to its instability in the gastrointestinal (GI) tract and its low permeability through the intestinal mucosa, requiring nonoral routes of delivery [2]. Moreover, oral administration of insulin would be directly channeled from the intestine or colon to the liver to avoid peripheral hyperinsulinemic effects [3]. For successful protein absorption from GI route, both the proteins and peptides must overcome several barriers. Initially, they have to overcome the destructive acidic pH in stomach. Secondly, they have to be protected from the intensive proteolytic enzyme activity of the intestine, and, finally, they have to pass through the intestinal epithelial cells which prevent transport of macromolecules due to their structural properties [4]. Consequently, researchers have used different strategies for manufacturing tablets or developing drug carrier systems capable of controlling drug delivery after oral administration.

 Recent researches have determined that polymeric compounds are useful carriers for high molecular weight drugs [5]. Biodegradable polymers such as chitosan have been used extensively in biomedical fields in the form of sutures, wound covering and as artificial skin. Deacetylation of chitin, the second most abundant biopolymer isolated from insects, crustacea such as crab and shrimps, as well as fungi, leads to poly(*β*-1,4-D-glucosamine) or so called chitosan [6]. Chitosan with excellent biocompatible and biodegradable properties has been used extensively in the pharmaceutical industry as drug delivery system [7]. Moreover, chitosan has been extensively investigated for its potential as a permeation enhancer across intestinal epithelium for peptides and proteins [8]. The mucoadhesive property of chitosan is mediated by its ability to spread over the mucus layer and additionally its positive ionic interactions with the negative charges of the cell surface membranes [9]. 

 Arabic gum (Acacia), a biocompatible and biodegradable polymer, is mainly used in oral and topical pharmaceutical formulation as a suspending and emulsifying agent [10]. It is also used in the preparation of lozenges and as a tablet binder. Arabic gum has also been evaluated as a bioadhesive in novel tablet formulations and modified release tablets [10]. 

 In the last decades, many strategies have been developed to enhance oral protein delivery [11]. Among these approaches, nanoparticulate systems have attracted especial interests for the following reasons. Firstly, nanoparticles are able to protect active agents from degradation [12]. Secondly, they can improve the drug transmucosal transport [13] and transcytosis by M cells, and thirdly, the particulate systems can provide controlled release properties for encapsulated drugs [14]. In recent years, ion gelation or polyelectrolyte complex formation (PEC) has drawn increasing attention for producing nanoparticles containing peptides [15]. The nanoparticles prepared by this method have several characteristics favorable for cellular uptake and colloidal stability, including suitable diameter and surface charge, spherical morphology, and a low polydispersity index indicative of a relatively homogeneous size distribution [16]. In addition, this method has the advantage of not necessitating aggressive conditions such as the presence of organic solvents and/or sonication during preparation; therefore, minimizing possible damage to proteins and peptides during ion gelation formation [17]. 

 This study deals with in vitro insulin release and ex vivo studies on intestinal section of sheep to investigate the permeation of insulin in free form and in nanoparticle form.

## 2. Experimental

### 2.1. Materials

Chitosan (95% deacetylated with viscosity of 1% W/V solution, 30 mPa.s) was purchased from Primex (Siglufjordur, Iceland). Crystalline recombinant human insulin (28.3 IU/mg) and Arabic gum were purchased from Eli-Lilly (Suresnes, France) and Arthur Branwell (Braintree, Essex, England), respectively. The other materials of pharmaceutical and analytical grades were used as received.

### 2.2. Preparation of Insulin Nanoparticles

The preparation of insulin nanoparticles was performed by a method previously set up in our laboratory [18]. Briefly, Known amounts of chitosan were dissolved in 0.1% of acetic acid solution to obtain define concentration under stirring at room temperature. In the second step, Arabic gum was dissolved in water to obtain a known concentration under magnetic stirring at room temperature. Nanoparticles were prepared by adding Arabic gum solution dropwise to chitosan solution containing insulin under gentle magnetic stirring (200–300 rpm) at room temperature. Nanoparticles were recovered by centrifugation at 4°C and 14,000 rpm for 15 min, and the supernatant was used for measurement of free Insulin by HPLC chromatography (HPLC, youngling, SDV SOS, Anyang, South Korea).

 The best formulation was selected using a 2^3^ factorial design experiment (Table 2) and was further used for in vitro release studies as well as ex vivo permeation studies.

### 2.3. Characterization of Insulin Nanoparticles

Mean diameter, polydispersity index (PDI), and Z-potential of insulin nanoparticles were measured using zeta sizer apparatus (Malvern, Z-S, Worcestershire, UK), and the range of polydispersity index between 0-1 was determined. Nanoparticles were analyzed with transmission electron microscopy (TEM, Phillips 400, KW 80, Eindhoven, and Holland). 

### 2.4. Insulin Association Efficiency

 Briefly, the insulin association efficiency was measured by HPLC method. The amount of insulin encapsulated in the nanoparticles was calculated by measuring the difference between the total amounts of the insulin added in the nanoparticle preparation solution and the amount of nonentrapped insulin remaining in the clear supernatant after the centrifugation. 

 The insulin loading level in nanoparticles was calculated based on the amount of insulin in the nanoparticles that was determined by measuring insulin in the HCl medium after filtering through a 0.1 *μ*m syringe filter by HPLC method [18].

### 2.5. In Vitro Release Study

Insulin release from nanoparticles was done in three different pH values of 0.1 N HCl, phosphate buffer solution (PBS) pH 6.5 and 7.2 in a 25 mL tube at 37°C and 75 rpm. Samples were withdrawn at predetermined time intervals and filtered through a 0.1 *μ*m filter. The filtrates were analyzed for the amount of insulin using HPLC method.

### 2.6. Ex Vivo Study

**Figure 1:**
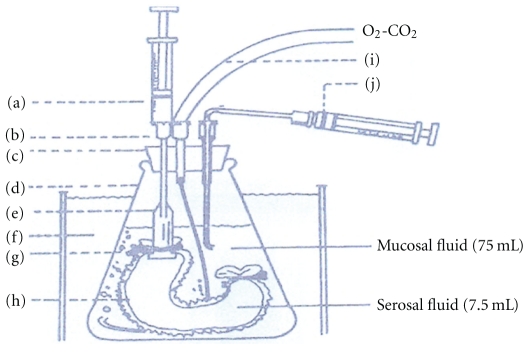
Apparatus used in the ex vivo studies in intestine: (a) disposable syringe for collection of serosal fluid, (b) hypodermic needle, (c) rubber stopper, (d) conical flask, (e) polyethylene centrifuge tube, (e) water bath (37°C), (g) tape used to fasten intestine to tube, (h) intestine, (i) mixture of gas inlet (O_2_ 95% and CO_2_ 5%), and (j) disposable plastic syringe used to collect mucosal fluid.

The procedure used is a modification of Barr and Riegelman method with some adjustments [19]. At first a section of intestine (about 7 cm) was removed from a male sheep under Phenobarbital anesthesia and washed with Krebs-Ringer bicarbonate solution, pH 7.4. The lumen was inverted with a glass rod and a tube was inserted in one side of the intestine and tied securely with tape. The other side of the intestine was tied and 1mL of Krebs-Ringer bicarbonate solution was poured through the hypodermic needle in the tube. The lumen of intestine was placed in a medium standard with 95% O_2_, 5% CO_2_ in phosphate buffer solution pH 6.5 at 37°C. In absorption studies, O_2_ and CO_2_ mixture was bubbled into the intestinal to obtain intestinal peristaltic movement (Figure 1). In certain periods of time samples with known volume were collected from the medium and were assayed by HPLC method, and the amount of insulin was calculated.

## 3. Results and Discussion

The insulin nanoparticles were prepared by ion gelation method using the electrostatic interactions between positively charged chitosan and negatively charged Arabic gum. In our previous study, various formulations (F_1_–F_8_) were prepared using 2^3^ factorial design experiments [18]. According to the results of factorial design experiments, three formulations (Table 2) were selected. The association efficiency (AE) of the nanoparticles was investigated in this study.

**Figure 2:**
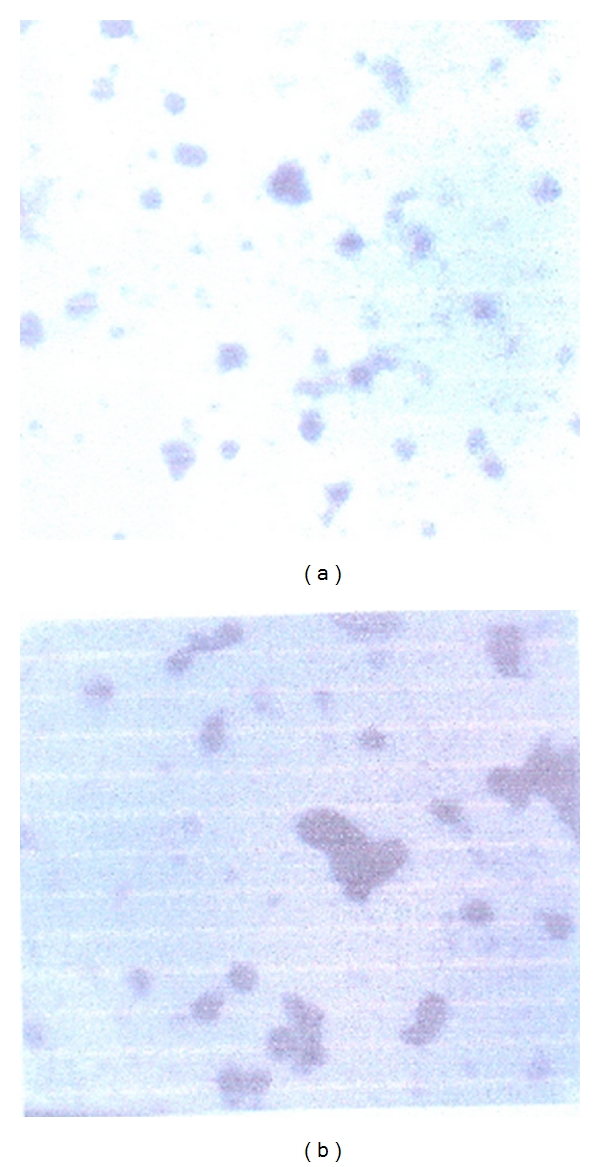
TEM micrographs of insulin nanoparticles for formulation (a) F_2_ and (b) F_3_.

 Moreover, the electrostatic interactions between the positively charged group of chitosan and negatively charged groups of Arabic gum plays an important role in the association efficiency of insulin in nanoparticles. These studies have shown that increasing the concentrations of chitosan and Arabic gum or the amount of insulin used in nanoparticle preparation are important factors in AE (Table 2) [18].  Figure 2 shows the TEM photograph of the nanoparticles. The nanoparticles showed spherical or oval shapes with relatively smooth surface. According to these figures and the results obtained from the zeta potential studies, the positive charges on the surface of nanoparticles could prevent the aggregation process.

 The particle size of nanoparticles is presented in Table 3 for the three selected formulations. The mean diameter of particles is 191, 172, and 177 nanometers for formulations F_1_, F_2_, and F_3_, respectively, and exhibited relatively narrow particle size distribution, as indicated by a relatively low polydispersity index values (Table 3). Zeta potential is used to determine the surface charge of nanoparticles [20]. Chitosan nanoparticles are all positively charged as shown in Table 3. This could be related to the type of particle formation mechanism between positively charged amine groups of chitosan that are neutralized by their interaction with negative charge of Arabic gum polymer. The residual amine groups would be responsible for the positive zeta potential. This effect may be related to the absorption of anionic groups by the long amino groups of chitosan to keep the high value of the electrical double layer thickness which in turn prevents the aggregation [21].

**Figure 3:**
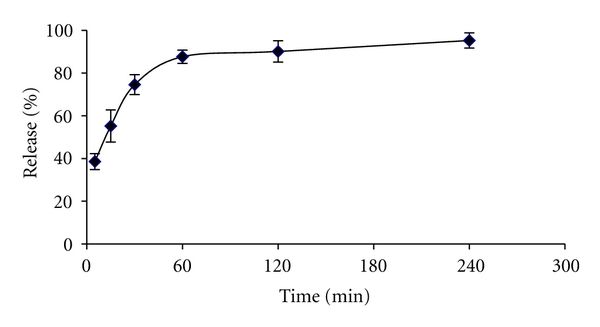
In vitro release profile of insulin from chitosan nanoparticles in HCl, pH 1.2.

**Figure 4:**
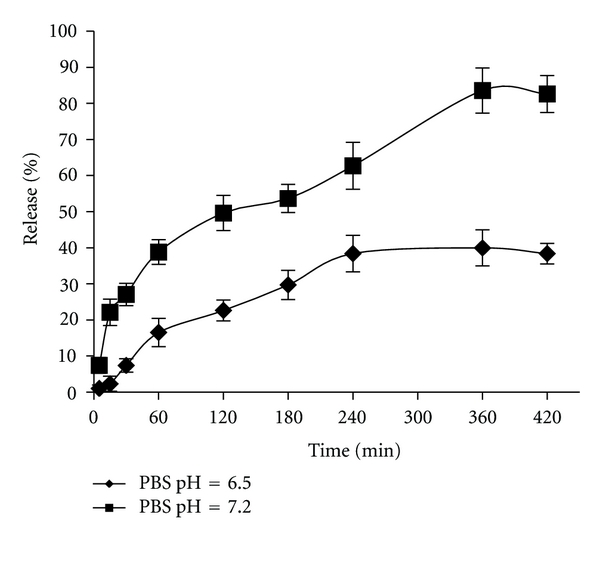
In vitro release profiles of insulin from chitosan nanoparticles in phosphate buffer solution, pH 6.5 and pH 7.2.

 The insulin release of nanoparticles was studied as a function of time for formulation F_3_. Figures 3 and 4 have shown the release profile of insulin in three different pH medium HCl 0.1 N, PBS pH 6.5, and pH 7.2 [18]. A burst effect of insulin release in acidic medium is related to high solubility of both chitosan and insulin (Figure 3). Furthermore, it is fair to say that a lot of insulin molecules are loosely attached on the surface of nanoparticles, and therefore insulin tends to easily move out and diffuse to the external medium.

 As shown in Figure 4 the release profile of insulin from the nanoparticles is different in higher pH values of 6.5 and 7.2. Moreover, the solubility of chitosan and insulin in PBS is lower than acidic medium and therefore a burst effect was not observed. The difference in insulin release at PBS pH 6.5 and 7.2 is probably related to the presence of Arabic gum. Arabic gum has a pH value of 4.5–5.0 in 5% W/V aqueous solution and therefore the polymer chains may swell in pH mediums higher than 6.5 and obtain more porosity in nanoparticle structures, resulting in more insulin release. For more studies, Fickian and nonFickian behavior have been used for determining the mechanism of drug release. 

 Equation *M*_*t*_/*M*_*∞*_ = *kt* ^*n*^ was used, where *M*_*t*_ is the amount of released insulin in a given time, *M*_*∞*_ is the total amount of insulin within nanoparticles, *k* and *n* are the equation constants, and *t* is the time.  Table 4 has shown the result for kinetic constant, diffusional constant, and type of release mechanism in formulation F_2_ and F_3_. The value of 0.823 for formulation F_2_ and 0.494 for formulation F_3_ are related to nonFickian transport [18]. The result suggests that transport is possibly controlled by diffusion and/or swelling of the polymer chains.

**Figure 5:**
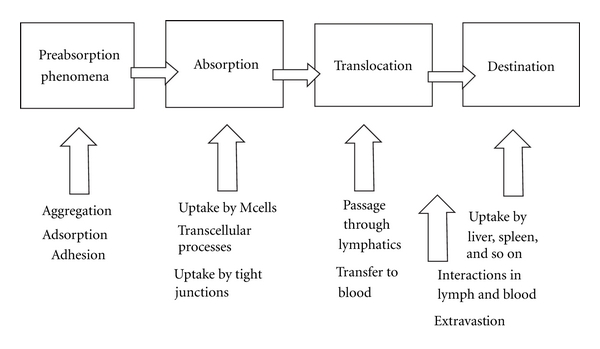
A simplified schematic of preabsorption and postabsorption processes in nanoparticle-dependent drug delivery to gastrointestinal sited following oral administration, highlighting the variety of processes involved in the journey of a nanoparticle from delivery to target.

 Today, the uptake of nanoparticulate systems through the gastrointestinal tract is a well-known and accepted phenomenon, and excellent reviews of the intestinal uptake of particles have been published. The bioactive molecules are transported into the GI by carriers with specific physicochemical characteristics that have no effect on the drug.  Figure 5 shows the simplified schematic of preabsorption and postabsorption processes in nanoparticle dependent drug delivery to gastrointestinal sites following oral administration.

**Figure 6:**
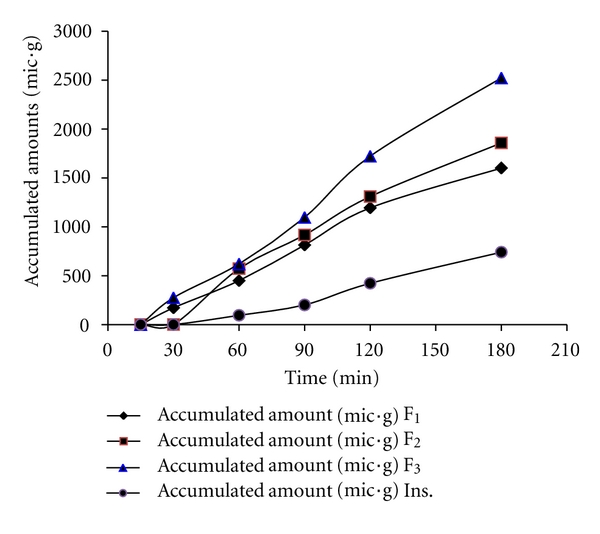
Profile of the amount of insulin absorption as function time for F_1_ (♦), F_2_ (■), and F_3_ (▲) nanoparticle formulations as well and as free insulin (*⚫*).

 The amount of insulin transported across the intestine barrier was measured by using modified gut sac method. Permeation profiles of insulin from formulation F_1_, F_2_ F_3_ as well as free insulin samples are depicted in Figure 6. It is clearly demonstrated that the permeation of insulin in the serosal medium in nanoparticulate form is much more enhanced in comparison with free-soluble insulin. Moreover, insulin is a macromolecule with hydrophilic properties that is not easily transported across the cellular membrane. 

 Nanoparticulate systems facilitate the transport of insulin through the intestinal barrier due to their protective effect as well as their charge properties. For the nanoparticles to be able to be permeated via the gut-associated lymphoid tissues the physical characteristic of the nanoparticles such as size, shape, specific surface, surface charge, and chemical stability are important factors to be considered. Moreover, the potential interactions with gut contents, transit time through the GI, transport through the mucosa, adhesion to epithelial surfaces, and aggregation of the particulates in contact with the fluid content of the gut must be considered. Some phenomena, such as aggregation, adsorption, and adhesion, can alter the zeta potential, hydrophilicity, and size of the nanoparticles. The zeta potential and mean diameter for F_1_, F_2_, and F_3_ formulations are close to each other so we cannot show the significant difference between these formulations (*P* < 0.05). In the intracellular pathway, it seems that nanoparticles regardless of their particle size are able to move through the cellular membrane via transcytosis or endocytosis mechanisms.

In intercellular pathway, the presence of tight junction between adjacent epithelial cells is the main barrier to the passage of macromolecules and hydrophilic agents such as peptides and proteins. The presence of the positive charge on the surface of the nanoparticles may interact with the actin filaments of intestinal epithelium disrupting the structure of the tight junctions and enabling the permeability of insulin through these junctions.

## 4. Conclusions

The main goal of administration of nanoparticles by the oral route is to lower the dose of the drug (and consequently to diminish its toxicity) as well as to improve patient compliance and supply an easy administration route. Other aims may be to decrease the fed/fasted variability and patient-to-patient variability. Efficient incorporation of bioactive molecules in nanoparticles requires in-depth study. 

 Hydrophilic nanoparticles based on chitosan are receiving increased interest as they could control the rate of drug release, prolong the duration of the therapeutic effect, and deliver the drug to specific sites in the body. 

 In this study, we developed a new nanoparticle system with Arabic gum for oral delivery of insulin. The effect of different variables on nanoparticle preparation was investigated. Results have indicated a small size, positive charge, and median AE for nanoparticles. It was shown that the release of insulin from nanoparticles was obtained with more than one mechanism (possibly diffusion, dissolution, and relaxation of the polymer chains). 

 Results showed that chitosan nanoparticles are able to enhance permeation of insulin as hydrophilic models through intestine epithelium. Indeed, our preliminary studies have shown that all the nanoparticles formulations are able to increase the amount of insulin transported across the intestine barrier compared to free insulin sample. Understanding the structure and function of bilayer of cell membrane and proteins that are involved in the formation of the intercellular junctions may create a new field for developing different mechanisms of drug permeation. Further investigations are required and are in progress in our laboratory to fully characterize and optimize these systems.

## Figures and Tables

**Table 1:** Parameters used in the factorial design experimental.

Factor	Low level	High level
(*x*_1_) Chitosan concentration (mg/mL)	1	10
(*x*_2_) Arabic gum concentration (mg/mL)	1	5
(*x*_3_) Insulin amount (mg)	5	10

**Table 2:** Three types of selected insulin nanoparticles based on 2^3^ factorial design experiment. *x*_1_, *x*_2_, and *x*_3_ are chitosan concentration (mg/mL), Arabic gum concentration (mg/mL) and insulin amount (mg) respectively, and association efficiency (run in triplicate) was selected as the dependent variable (*y*).

Formulation code	*x*_1_	*x*_2_	*x*_3_	*y*% (mean ± sd) (*n* = 3)
F_1_	10	1	5	31.2 ± 2.83
F_2_	10	5	5	35.8 ± 4.31
F_3_	10	5	10	37.5 ± 2.75

**Table 3:** Characteristic of the nanoparticles containing insulin for different formulations F_1_–F_3_.

Formulation code	Mean diameter (nm, *n* = 3)	Polydispersity	Zeta potential (mv)	AE (%)	Loading capacity
F_1_	191 ± 17	0.48	42.6 ± 3.4	31.2 ± 2.83	11.8 ± 0.56
F_2_	172 ± 10	0.26	41.7 ± 2.9	35.8 ± 4.31	13.73 ± 0.43
F_3_	177 ± 10	0.25	40.5 ± 3.3	37.5 ± 2.75	16.28 ± 0.78

**Table 4:** Kinetic constants (*k*), diffusional (*n*) and determination coefficient (*r* ^2^) determined by the linear regression of Ln(*M*_*t*_/*M*_*∞*_) against Ln *t*.

Formulation	*n* (*X* ± SD, *n* = 3)	*k* (*X* ± SD, *n* = 3)	*r* ^2^
F_2_	0.823 ± 0.0341	0.0008 ± 0.0001	0.956
F_3_	0.494 ± 0.0268	0.0019 ± 0.0003	0.958
